# Geographical and Ethnic Differences Influence Culturable Commensal Yeast Diversity on Healthy Skin

**DOI:** 10.3389/fmicb.2019.01891

**Published:** 2019-08-27

**Authors:** Cheryl Leong, Bettina Schmid, Min Jet Toi, Joyce Wang, Antony Sagayaraj Irudayaswamy, Joleen Peh Zhen Goh, Philipp P. Bosshard, Martin Glatz, Thomas L. Dawson

**Affiliations:** ^1^Agency for Science, Technology and Research (A^∗^STAR), Skin Research Institute of Singapore, Singapore, Singapore; ^2^Department of Dermatology, University Hospital Zürich, Zurich, Switzerland; ^3^Faculty of Medicine, University of Zürich, Zurich, Switzerland; ^4^Center for Cell Death, Injury & Regeneration, Departments of Drug Discovery & Biomedical Sciences and Biochemistry & Molecular Biology, Medical University of South Carolina, Charleston, SC, United States

**Keywords:** skin, commensal yeast, *Malassezia*, geography, ethnicity, fungi

## Abstract

Commensal fungi such as *Malassezia*, *Candida*, and *Rhodotorula* are common on healthy skin but are also associated with opportunistic invasive and superficial infections. Skin microbial community characterization has been extensively performed worldwide, with a focus on the 16S bacterial community. These studies have focused on geographically distinct or targeted cohorts with variable reported species distributions of commensal yeast species. To determine the effects of extrinsic environmental factors such as geography, climate, and ethnicity on detected healthy skin commensal yeast diversity, we compared cohorts from Singapore and Zürich, Switzerland, representative of two geographically and climatically distinct regions comprising multi-ethnic (Chinese, Malay, Indian, Caucasian) and predominantly white Caucasian cohorts, respectively, using identical skin sampling and culture methods. We chose to use a culture-based approach as cultures isolated from patients are still required for studies of pathogenicity and antifungal susceptibility. Detection of yeast species by culture-dependent and independent sequencing-based methods suggest healthy skin diversity reflects a species distribution representative of the geography, climate and ethnic background of their local populations. Culture success and species diversity was also found to be dependent on climate, with warm tropical climates favoring high positive culture rates and greater species diversity. Multilocus sequence typing data suggests some strains are geographically distinct and may be used to segregate potential disease-causing commensals. For accurate collection and characterization of skin microbial communities, it remains recommended to employ a combination of culture-dependent and sequence-based culture-independent methods. Characterization of healthy mycobiomes in geographically distinct local populations will be useful in defining the role of commensal fungi in health and disease.

## Introduction

Yeasts are an integral part of the skin microbial communities and play an important role in skin health and disease (Findley et al., 2013). *Malassezia*, a lipid dependent yeast, is the dominant resident fungal species in human adults and accounts for up to 80% of skin fungal genomes (Gao et al., 2010). Of the 18 presently known species, up to 10 are found on human hosts and are associated with superficial mycoses such as pityriasis versicolor or seborrheic dermatitis (Theelen et al., 2018). Other common skin yeasts include *Candida* and *Rhodotorula*, with the former being the most common cause of invasive fungal infections worldwide (Richardson and Lass-Flörl, 2008). Reduced skin microbial diversity is associated with increased incidence and severity of skin disease (Baviera et al., 2014). Studies suggest that many infectious *Candida* and *Malassezia* strains have commensal origins (Chang et al., 1998; Kam and Xu, 2002). This underscores the potential role of commensal yeasts as opportunistic pathogens. Culture-independent genomic methods (e.g., species-specific PCR/qPCR, next generation sequencing) are commonly used for the clinical detection and molecular differentiation of commensal yeast species(Gupta et al., 2000; Sugita et al., 2001; Gaitanis et al., 2002; Paulino et al., 2006; Zhang et al., 2013; Jagielski et al., 2014). However, culture-dependent methods are still required for studies of pathogenicity and antifungal susceptibility (Leong et al., 2017), hence our culture-based approach.

Variations in reported species distribution in global healthy and diseased cohorts have confounded our understanding of the role of *Malassezia* in health and disease. Epidemiological studies on the role of these yeasts in opportunistic infection suggest that in addition to variations in culture and sampling methods, factors such as host susceptibility (Grice and Dawson, 2017) and other extrinsic environmental factors may influence their prevalence and species distribution (Kim et al., 2018). These include factors such as phylogeography, climate, ethnicity and inter-individual cultural, and lifestyle variations (Duarte et al., 2003; Gaitanis et al., 2009; Akaza et al., 2012; Pappas et al., 2013). Genetic fingerprinting for *Malassezia furfur* has shown evidence of environment being a driver by revealing genetic variation associated with related diseases, host ethnicity and geographic origin (Gupta et al., 2004; Gaitanis et al., 2009; Zhang et al., 2010; Pedrosa et al., 2019). From culture-based studies, *Malassezia sympodialis*, *Malassezia globosa*, and *Malassezia restricta* are most commonly reported in the Northern Hemisphere whereas *M. furfur* is most commonly reported in the Southern hemisphere (South America). There is limited information about the genetic diversity and global genotype distribution of non*-Candida* commensal fungi, largely due to fungal banks being derived from selected geographical locations or cohorts.

To address these factors, we compared two cohorts of healthy individuals from two geographically and climatically distinct regions. Singapore is an island city in south east Asia with a tropical climate [Mean temperature: 23–30°C, Relative average humidity range: 80–85% (World Weather and Climate Information©, 2010–2019)]. The multi-racial demographics of Singapore’s population (Chinese, Malay, Indian, Caucasian) is ideal for skin ethnicity comparisons. In contrast, the canton of Zürich, (northern Switzerland), boasts a moderately continental climate with four distinct seasons and a predominantly Caucasian population (Mean temperature range: 4–12°C, Relative average humidity range: 72–85%) (World Weather and Climate Information©, 2010–2019). We hypothesize that geographical climate-associated differences, skin ethnicity, and inter-human relationships affect the yeast carriage and genotype distribution. These factors may account for the differences in globally reported fungal yeast species.

## Materials and Methods

### Cohort Characteristics

We obtained swabs from two body sites (left lateral side of the nose and/or scalp) of healthy volunteers seen at the Skin Research Institute of Singapore, Singapore. 48 subjects were sampled on the side of the nose/aral crease and 44 subjects were sampled on the left temporal scalp. Sampling for both cohorts was performed separately (Supplementary Figure 1). For detailed yeast species characterization, gender and race comparisons from nasal skin swabs, only data from the 37 individuals from four major ethnic groups (10 Chinese, 7 Malay, 10 Indian, and 10 Caucasian) of equal gender distribution were analyzed. Subjects of mixed ethnicity were excluded in ethnically segregated species comparisons. Sampling was performed from December 2017 to March 2018.

Only one body site (left lateral side of the nose) was sampled using identical methods as above for 20 healthy volunteers (10 males, 10 females) seen at the Department of Dermatology at the University Hospital of Zürich, Switzerland as part of an epidemiological survey of healthy skin. For detailed yeast species characterization, only data from the 11 culture positive Swiss individuals were analyzed. Sampling for this cohort was performed in June–July of 2017.

All sampling was performed according the Declaration of Helsinki and approved by the respective ethics committees in Singapore (National University of Singapore, Institutional Review Board, NUS-IRB-237) or Zurich, Switzerland [Cantonal Ethics Commission (KEK), Zurich, KEK-ZH-NR: 2010-0117/0 and the Swiss Ethics Committees on research involving humans (BASEC), BASEC-Nr. 2016-00301]. Participants were between 22 and 65 years of age, having been living in Singapore/canton of Zürich, Switzerland for at least 2 weeks with no overseas travel and judged to be healthy based on the absence of overt skin diseases.

### Sample Collection

Briefly, sterile diagnostic swabs (COPAN Italia, Brescia, Italy or Fortuna Scientific, Singapore) were moisturized with saline and the whole left lateral side of the nose was swabbed for 10 s. Swabs were then directly inoculated onto modified Leeming Notman agar (mLNA) (Kaneko et al., 2007) plates containing 50 μg/ml of chloramphenicol and 100 μg/ml streptomycin (Sigma-Aldrich, Singapore) by streaking and rolling across the entire surface of the plate up for up to three times to maximize colony yield. Plates were incubated at 32°C for 2 weeks. No significant differences in sampling with different swab brands was observed (data not shown) and choice of swab was made based on availability in the respective geographical regions.

### Species Identification by Culture-Dependent Methods

Modified Leeming Notman agar plates were monitored over a course of 1–2 weeks for colony growth. Characteristics such as morphology, texture, color, speed of growth, and phenotype changes were recorded. After 2 weeks, up to 10 colonies of the same phenotype were picked for further culture expansion. Briefly, cultures were lysed in universal DNA extraction buffer (Goldenberger et al., 1995) containing 20 μg/ml of Proteinase K (Sigma-Aldrich, Singapore). The 18S ITS (ITS1-5.8S-ITS2) ribosomal region was amplified and sanger sequenced (Ciardo et al., 2006). Species identification was performed with the recorded species/strain having the highest BLAST score with a minimal match of 97%.

### Species Identification by Culture-Independent Methods

To compare the reproducibility of culture-dependent methods, the right lateral side of the nose (parallel to the side from which cultures were derived) was tape stripped 50 times using a D-Squame^®^ standard sampling disks (Cuderm^®^ Cooporation, Dallas, TX, United States). This was performed for four male Singapore subjects, one from each ethnicity. DNA extraction from each tape was performed using the MasterPure^TM^ Yeast DNA purification kit (Epicentre, United States) as per manufacturer’s instructions combined with bead beating with Lysis Matrix E (MP Biomedicals, Singapore) during the lysis step. Species-specific PCR amplification was performed as described (Zhang et al., 2013).

### LoD and LoQ Determination by Real-Time Quantitative PCR (RT-qPCR)

Amplified products of the ITS regions for each species were cloned into a pCR4.0 vector using TOPO TA Cloning Kit (Life Technologies, Singapore) as per manufacturer’s instructions. Real-time qPCR was carried out with the respective species-specific primers against each respective ITS plasmid standard across seven dilutions over the dynamic range of 3,000,000–3 ITS copies as quantified via Qubit dsDNA HS Assay Kit (Thermofisher Scientific, Singapore). Briefly, a 10 μl reaction mix was prepared containing 1 μl template, 5 μl of Luna Universal qPCR Mastermix (NEB, Singapore), 0.25 μl of each forward and reverse primer (10 μM), and 3.5 μl UltraPure water. Limit of detection (LoD) and limit of quantitation (LoQ) were calculated as described (Armbruster and Pry, 2008).

### Data Analysis

Positive culture rate was calculated by taking the percentage of the number of subjects whose swabs yielded yeast colonies on mLNA after 2 weeks in culture over the total number of subjects sampled. To compare the number of unique species identified on each subject, an unpaired, two tailed *t* test was performed. For quantitative comparisons of the diversity of species across different subjects, the Simpson’s Index, Shannon–Wiener Species Diversity Index and Evenness were calculated as follows: Shannon-Wiener Species Diversity Index, H = -SUM[(pi) × ln(pi)] E = H/H_*max*_ where, SUM = Summation pi = Number of individuals of species i/total number of samples S = Number of species or species richness H_*max*_ = Maximum diversity possible E = Evenness = H/H_*max*__._ Simpson’s Diversity Index = 1 - D E_1__/D_ = (1/D)/S Where, D = Simpson’s Index of Diversity S = Sum of numbers data.

### Multilocus Sequence Typing Analysis

Molecular typing was performed for isolates of *M. furfur*, *M. sympodialis*, *M. globosa*, *M. restricta*, and *Malassezia slooffiae* using the following loci – (1) Internal transcribed spacer regions (ITS1-5.8S-ITS2), (2) Intergenic Spacer Regions (IGS), (3) D1/D2 26S rRNA regions, (4) ß-tubulin, and (5) Elongation Factor-1 regions (Lorch et al., 2018). The number of strains analyzed was based on the minimum number of strains of each species available to us for analysis if not already available in GenBank. Sequencing of PCR products was performed using the BigDye Terminator v3.1 Cycle Sequencing kit (Applied Biosystems, United States). These sequences were pooled with locus-specific sequences available on the GenBank database^1^. Sequence alignments were performed using the Multiple Sequence Alignment tool (Corpet, 1988) and/or the software Clustal Omega^2^. Strain information from non-local isolates was sourced from prior publications (Theelen et al., 2001; Gupta et al., 2004; Cabañes et al., 2005; Jagielski et al., 2014; Lian et al., 2014).

Duplicate loci sequences from multiple strains were identified by ElimDupes^3^ and removed from further analysis. Phylogenetic tree construction and bootstrap analysis (1000 replicates) was performed using the Bionumerics 7.6 software package (Applied Maths, United States). Neighbor-joining, UPGMA, maximum-parsimony and maximum-likelihood methods were used for phylogenetic tree construction (Saitou and Nei, 1987; Felsenstein, 1988, 1996). Multiple correspondence analysis (MCA) and hierarchical clustering analysis was performed using the “factoMineR” package R-version 3.4.3 (R Development Core Team, Vienna, Austria).

## Results

### *M. globosa*, *M. furfur*, *M. restricta*, *M. sympodialis*, and *Malassezia dermatis* Are Common in Singapore

**FIGURE 1:**
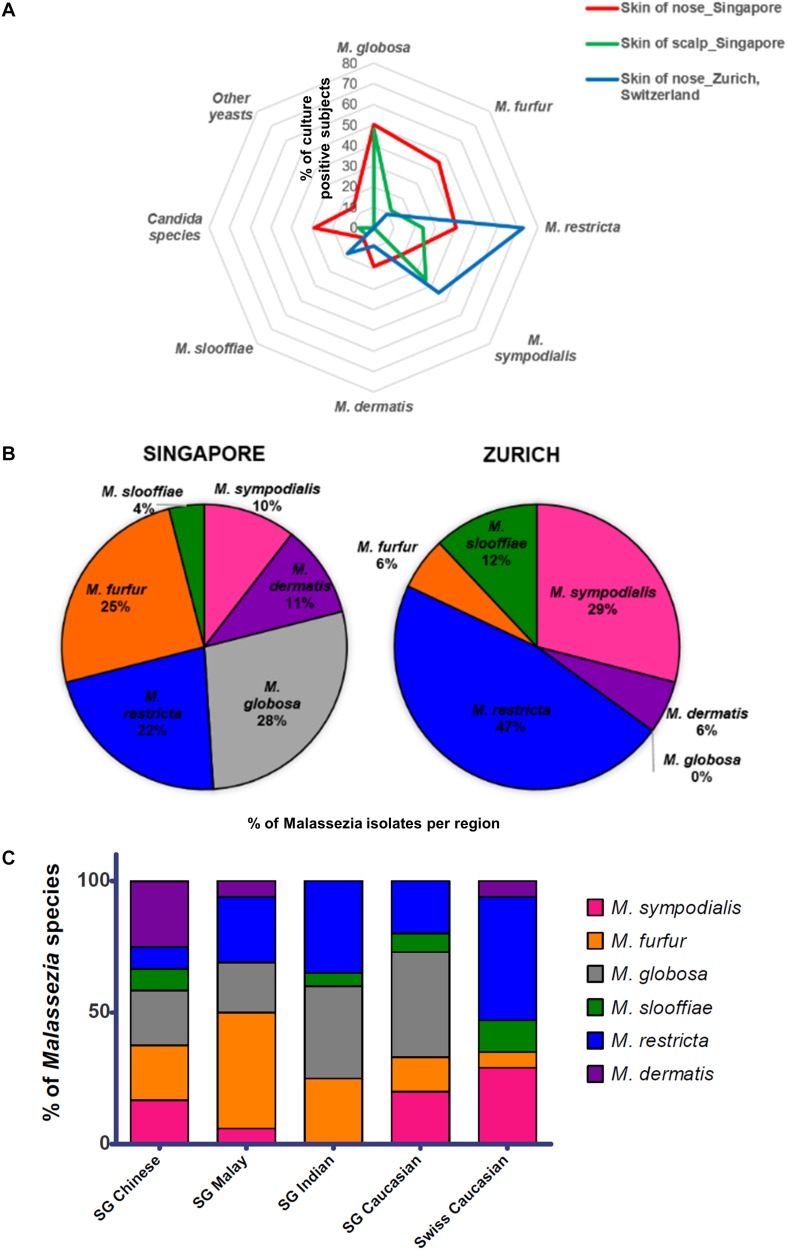
**(A)** Radar plot of percentage of subjects that were culture positive for each species (*y*-axis) as sampled from the respective cohorts and body sites, **(B)** pie charts showing percentage of each *Malassezia* species isolated from the skin of the nose calculated as a percentage of total *Malassezia* isolates from each respective cohort, and **(C)** *Malassezia* species isolated from the skin of nose of Singapore (*n* = 37, 10 Chinese, 7 Malay, 10 Indian, and 10 Caucasian, equal gender) and Swiss (*n* = 11) cohort subjects, represented as a percentage of the total *Malassezia* species isolated from each ethnic group.

Skin swabs from healthy Singapore subjects yielded a positive yeast culture rate of 87.5% (42 of 48 individuals) from the side of the nose and 95.5% (42 of 44 individuals) from the scalp. All positive cultures from swabs taken from the side of nose contained *Malassezia*. *M. globosa* (56.8%, 21 of 37 subjects), *M. furfur* (48.6%, 18 of 37), and *M. restricta* (40.5%, 15 of 37) were the dominant species in this Singapore cohort (Figure 1A and Table 1). Other *Malassezia* species included *M. sympodialis* 21.6% (8 of 37), *M. dermatis* 21.6% (8 of 37), and *M. slooffiae* 13.5% (5 of 37) (Figure 1A). *Candida* (e.g., *Candida parapsilosis*, *Candida orthopsilosis*) and *Rhodotorula* were cultured from 35.1% (13 of 37) and 13.5% (5 of 37) of the sampled subjects, respectively (Table 1). Fewer species of *Malassezia* were detected on the scalp, with *M. globosa* (47.6%, 20 of 42) and *M. restricta* (23.8%, 10 of 42) being the dominant colonizing species respectively (Figure 1A and Table 1). Other fungi such as *Aspergillus*, *Aureobasidium*, *Pallidocercospora*, *Cladosporium*, *Hortaea*, and *Trichosporon* were also observed sporadically in culture although their presence was not quantified.

**TABLE 1:** Fungal species and numbers isolated from the skin of healthy volunteers by culture-dependent methods.

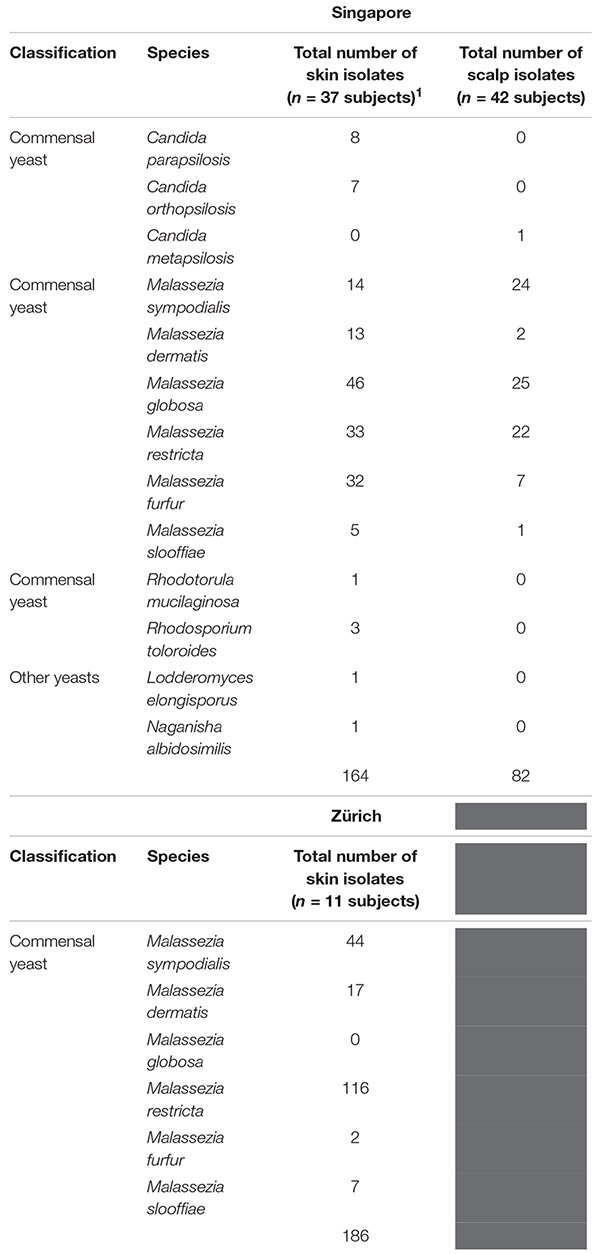

*^1^Species analysis was performed for 37 of 42 culture positive subjects as some subjects were excluded owing to mixed race background or contamination issues.*

### Swiss Samples Have a Lower Positive Culture Rate and Are Dominated by *M. restricta* and *M. sympodialis*

The rate of positive culture from skin swabs taken from the side of the nose of healthy Swiss individuals with the same sampling methods and culture conditions was 55.0% (11 of 20), which was lower than the observed positive culture rate in Singapore (87.5%). *M. restricta* and *M. sympodialis* were the dominant cultured yeasts (47 and 25% of Swiss *Malassezia* isolates (Figures 1A,B).

### *Malassezia* Species Distribution Is Influenced by Skin Ethnicity and Geographical Location

**FIGURE 2:**
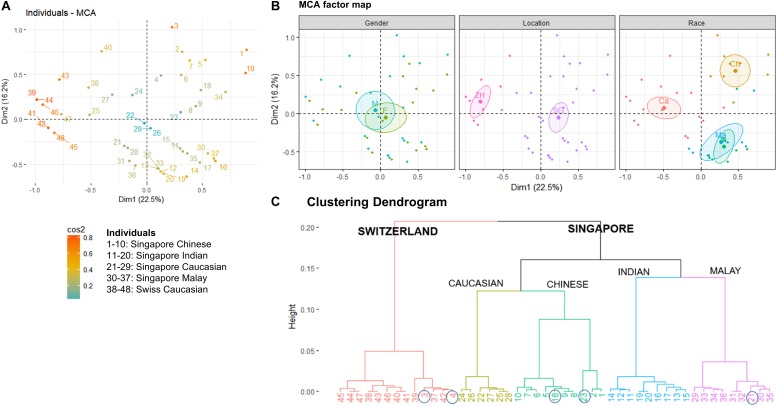
**(A)** Multiple Correspondence Analysis (MCA) plot showing the clustering of 48 individuals form Singapore and Zürich, Switzerland based on geography, gender, race, and *Malassezia* species cultured from the skin of the nose, **(B)** factor maps of individual clustering by gender (M – Male, F – Female), geography (SG – Singapore, ZH – Zürich), and race (Ch – Chinese, Ma – Malay, Ind – Indian, Ca – Caucasian) as defined by a 95% confidence ellipse, and **(C)** clustering dendrogram showing the clustering of individuals by hierarchical clustering. Numbering of individuals correspond to the legend in **(A)**. Circles indicate individuals that do not cluster according to their expected geography or race.

The average Singapore subject carries a higher number of *Malassezia* species compared to the average Swiss subject (2.03 species vs. 1.55 species, ^∗^*p* < 0.05), with greater species diversity and eveness (Table 2). Variations were observed in the species isolated from different ethnic groups (Figure 1C). *M. sympodialis*, *M. slooffiae*, and *M. dermatis* were selectively absent in some ethnic groups. The Singapore and Swiss Caucasian cohorts showed different species distribution profiles, with *M. globosa* being absent in the Swiss cohort and *M. restricta* being two times more commonly isolated on Swiss Caucasian skin compared to Singapore Caucasian skin (Figure 1B). Multiple correspondence analysis (MCA) was performed to identify individuals of similar profile and associations between the presence of different commensal yeast species. Comparison of the Singapore and Swiss cohorts revealed that the Swiss cohort formed a distinct cluster (Figures 2A,B), despite both cohorts having Caucasian individuals of European decent. The Singapore Chinese cohort also formed a distinct cluster. Singapore Malay and Indian cohorts were closely associated and the Singapore Caucasian cohort fell midway between the Swiss Caucasian cohort and the general Singapore cohort (Figure 2B).

**TABLE 2:** Species and genotype diversities of *Malassezia* skin isolates.

	**# of subjects**	**# of strains analyzed**	**Simpson’s Index**	**Shannon** **Index**	**Evenness**
Singapore	37	144	0.2186	1.65	0.92
Male	18	78	0.2201	1.58	0.88
Female	19	66	0.2094	1.57	0.88
Zürich	11	186	0.4518	1.31	0.81
Male	7	125	0.3846	1.13	0.7
Female	4	61	0.6612	0.64	0.58

No significant differences in detected species numbers were observed when comparing gender and age (*p* > 0.05, Figure 2B and Supplementary Figures 2, 3) and no *Malassezia* species was specifically correlated with any one ethnic group (Figure 1C and Supplementary Figures 2–4). Hierarchical clustering showed similar ethnic group and geographical clustering (Figure 2C), although some individuals were observed to fall outside of their ethnic group or geographical cluster (Figure 2C circled). Some categorical factors such as the presence of *C. orthopsilosis*, *M. globosa*, *M. slooffiae*, and *M. restricta* were observed to be associated with gender (Supplementary Figure 5). However, the inclusion of other commensal yeast species did not significantly change clustering (Supplementary Figure 5).

### Detection of Commensal Yeast Species by PCR

**FIGURE 3:**
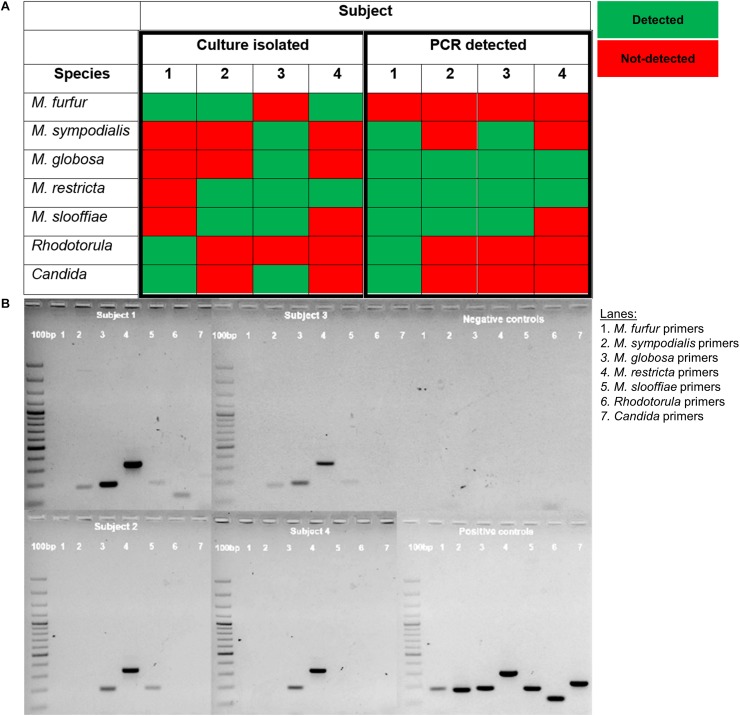
**(A)** Table comparison of commensal yeast species detection by culture-dependent and culture independent methods in four subjects and **(B)** PCR detection using species-specific primers of gDNA isolated from the parallel side of the skin of the nose from which cultures were swab-derived. gDNA isolated from reference strain cultures representative of common isolates of *Malassezia* (CBS 9369, CBS 7222, CBS 7966, CBS 7877, CBS 7956), *Rhodotorula* (ATCC 9449), and *Candida* (ATCC 22019) were used as positive controls. A 100 bp ladder (GeneRuler^TM^) was run on the leftmost column of each gel.

We compared PCR detection methods and culture-based results from tape strips obtained from opposite sides of the nose for four male volunteers representing each major ethnic group (Chinese, Malay, Indian, Caucasian). PCR methods were able to detect six of the seven tested species of commensal yeasts. *M. globosa* and *M. restricta* were the most robustly detected by PCR. The only exception was *M. furfur*, which was detected by culture only in three of the four subjects but not detected in any of the subjects by PCR (Figure 3). This unlikely to be a result of lower sensitivity as Limit of Detection (LoD) and Limit of Quantitation (LoQ) values for *M. furfur* primers were established to be the lowest of all five tested species-specific primers sets (Supplementary Table 1). These results suggest that there are limitations in the respective culture-dependent and independent species detection methods, which warrants further study.

### Multilocus Sequence Typing Identifies Geographic and Community Specificity

**FIGURE 4:**
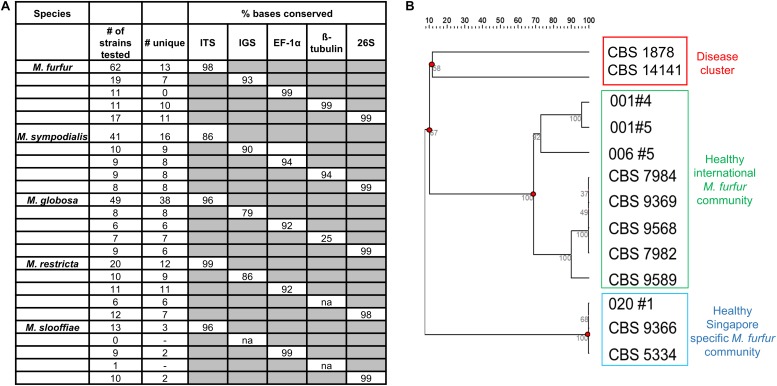
**(A)** Percentage conserved bases of five commonly isolated species of *Malassezia* across five loci (Internal transcribed spacer region – ITS, Intergenic spacer region (IGS), Elongation factor – 1α (EF-1α), ß-tubulin and the 26S ribosomal region) as tested across sequences derived from isolates collected and NCBI databases and **(B)** unrooted dendrogram derived from 13 unique ITS sequences of *M. furfur* identify healthy and disease clusters which are representative of strains isolated in Singapore and internationally. Sequences not represented by any known reference database ID are indicated by their subject/strain codes.

Five different loci – internal transcribed spacer region (ITS), 26S ribosomal region (26S), intergenic spacer region (IGS), Elongation Factor alpha 1 (EF-1α) and ß-tubulin were sequenced for six or more strains of *M. furfur*, *M. sympodialis*, *M restricta*, *M. globosa*, and *M. slooffiae* (Lorch et al., 2018). This includes reference strains in the CBS database, strains derived from primary isolates in Singapore and Zürich, and sequences derived from the NCBI sequence database. The 26S loci was 99% identical across all species of *Malassezia* and was not suitable for strain typing (Figure 4A). IGS, EF-1α and ß-tubulin were observed to be potential candidates for strain typing of *M. sympodialis*, *M. globosa*, and *M. restricta* (<99% conserved nucleotides). However, these loci could not be analyzed in all cases, as the primer amplification did not work well for all species despite extensive PCR optimization.

For *M. furfur* and *M. sympodialis*, ITS profiles provided the best resolution relative to other loci in their ability to enable the visualization of clear groups or clusters of strains. Based on data describing isolate backgrounds, we observed segregation of disease derived *M. furfur* isolates (CBS 1878 and CBS 14141) from healthy derived *M. furfur* isolates (Figure 4B). Other *M. furfur* isolates from healthy individuals were generally well represented in the international community (Singapore and Zürich), with a small subgroup found mainly in Singapore. We were unable to infer any phylogeographical or healthy/disease isolate clustering on dendrograms derived from ITS strain typing with *M. globosa* and *M. sympodialis* (Supplementary Figures 6A,B) due to limited information on the background of database strains (unclear where they were isolated or if they were from healthy or diseased skin). Some *Malassezia* strains were observed to be inter-individual or community specific, with strains from the same species being found only on certain groups of individuals. A single healthy host was observed to carry up to two to three culturable *Malassezia* species, often with multiple strains of the same species (Supplementary Figure 6B, Subject 031).

## Discussion

By comparing two cohorts of healthy individuals based in Singapore and Zürich, Switzerland using identical skin sampling and culture methods, we have observed that diversity in commensal skin fungi varies with geography and ethnicity. The hot, wet, tropical Singapore climate likely facilitates a higher skin fungal load resulting in high positive culture rates (80–90%), with individuals living in Singapore carrying an average of two to three fungal species as detected by optimized culture-dependent methods and two to six species as detected by optimized culture-independent methods. In some instances, saprophytic fungi such as *Aspergillus* and *Aureobasidium* were detected in culture. However, these species are not known to be commensal and likely to be a result of environmental contamination. Subjects from the temperate Switzerland climate had lower positive culture rates (55%), with the average individual carrying only one to two culturable species. Geographical climate factors such as lower temperature and humidity seem likely to account for a lower microbial load on skin and hence lower culturomic detection. *M. globosa*, which was missing in Swiss samples, has previously been identified to grow preferentially at higher temperatures and in the presence of sweat compounds, which are similar to conditions on the skin in hot summer conditions (Akaza et al., 2012) and the standard growth range of *Malassezia* (28–35°) (Lorch et al., 2018) is generally suited to warmer climates.

There is some evidence from results in the literature (Theelen et al., 2001; Gupta et al., 2004) that some disease-related isolates may cluster together, e.g., PM and JLPK series isolates. It is also possible that not all disease isolates are the disease-causing pathogens and some isolates could be commensals acquired during sampling. ITS strain typing of the six main culturable species (*M. furfur, M. sympodialis, M. globosa, M. restricta, and M. slooffiae*) showed no strict ethnic group segregation. The different *Malassezia* species observed across different ethnic groups and genders suggest that there are additional factors contributing to healthy skin microbial diversity. These may include age and ethnic variations in sebaceous lipids (Pappas et al., 2013), which may indirectly influence the species of colonizing lipid dependent *Malassezia* yeast. However, the possible impact of age on skin microbial diversity was not apparent as our study was designed largely to address geographical and ethnic differences.

A culture-dependent approach was employed for this study on the premise that the conversion of commensal yeasts into opportunistic pathogens requires the ability to replicate outside their primary host environment. Cultures are also required for comprehensive genomic analysis, strain typing and are be valuable for further antifungal susceptibility and physiological assay testing as they may reflect antifungal usage patterns across different geographic areas. These parameters presently cannot always be addressed by Next-Generation-Sequencing (NGS) methods due to limited depth and read numbers as well as the lack of comprehensive strain level databases. However, significant limitations still exist in the standardization of skin microbial sampling protocols. It remains difficult to control for inter-individual swabbing techniques (e.g., how hard or evenly the subject is swabbed) despite published standardized swabbing and culture protocols. Choice of swabbing region such as the nasal crease of the nose and the scalp may also be affected by the types of cosmetic products used (e.g., sunscreen, make-up, cleansers, shampoos) and other lifestyle habits, which are extremely difficult to control (and were not controlled in this study). We also did not take into account the extended duration over which subjects have resided in their respective geographies (Singapore and Zürich). Thus, subjects who have only been living in Singapore for a few months to a year may still have skin microbial communities more reflective of their original geographies. In view of the small size, border geographical locations and large foreign populations [40% Singapore (Department of Statistics Singapore, 2018), 32% Zurich (Stad Zürich, 2019)], and also taking into account that Swiss and Singapore subjects travel frequently across the border to nearby South-East Asian countries or European countries, we opted not to have strict inclusion criteria for travel as excluding subjects who travel beyond a 2-week period would further reduce the cohort size to be unusable and affect the external validity of our study (Rothwell, 2005, 2006). It is also extremely rare to identify individual volunteers who have lived their whole life in one location. We believe that a “normal microbiome” would also include the possibility of a subject traveling outside of his home residence for short periods.

More fastidious *Malassezia* such as *M. globosa* and *M. restricta* are difficult to grow and isolate, possibly due to their low efficiency of colony formation. To address the possibility of differential growth rates affecting the detection of slower growing fungal species in culture, we performed species-specific PCR detection using fungal gDNA isolated from tape strips taken in parallel to our culture swabs. PCR showed higher sensitivity to detect *M. globosa* and *M. restricta* even when they were not detected in culture (data not shown). However, PCR failed to detect *M. furfur*, which is one of the key *Malassezia* species involved in opportunistic systemic infection and superficial fungal infection. This could be due to the lower DNA yield for *M. furfur* compared to other *Malassezia* species or the high phylogenetic diversity of the species, which may affect the efficiency of primer detection.

Multilocus sequence typing (MLST) and Amplified Fragment Length Polymorphism (AFLP) are complementary analysis methods which may be used for strain typing to provide or extend epidemiological information (Mietze et al., 2011) and have been established to show good agreement for *Malassezia* (Restrepo et al., 2018). In this study, we have opted to use MLST as our preferred analysis method due to its ability to provide unambiguous and reproducible sequencing data which is easily stored and shared on online databases (Parisi et al., 2010). As our analysis was based on our local (Swiss and Singapore) derived strains which are not yet available in public databases, we would expect to see differences in segregation when using different data sources. The building of more diverse and well-annotated fungal cell line banks, fungal genome databases and fungal reference strains reflecting greater ethnic, geographical and cohort (e.g., healthy and disease isolates) diversity is also critical for making valuable inferences on strain typing clustering patterns. While we have observed interesting strain type clustering for some loci, we are presently unable to infer any useful information from these patterns due to a lack of knowledge on the background of some strains, with the exception of *M. furfur*, the most well characterized *Malassezia* species (Theelen et al., 2001; Gupta et al., 2004).

In summary, we have performed a comparison of the commensal yeast species present on the skin of two geographically and ethnically distinct cohorts of healthy individuals. Up to six species of *Malassezia* as well as *C. parapsilosis* and *Rhodotorula* were detected by culture-dependent and independent methods, with individuals from Singapore yielding a higher positive culture rate and carrying more commensal yeast species than individuals from Zürich, Switzerland. MLST data suggests that some strains are geographically distinct and may be used to segregate potential disease-causing commensals. For accurate collection and characterization of skin microbial communities, it remains recommended to employ a combination of culture-dependent and sequence-based culture-independent methods. Characterization of healthy mycobiomes in geographically distinct local populations will be useful in defining the role of commensal fungi in health and disease.

## Data Availability

The raw data supporting the conclusions of this manuscript will be made available by the authors, without undue reservation, to any qualified researcher.

## Ethics Statement

All sampling was performed according the Declaration of Helsinki and approved by the ethics committee in Singapore (NUS-IRB-237) or Zurich (KEK-ZH-NR: 2010-0117/0 and BASEC-Nr. 2016-00301), with written informed consent from all subjects.

## Author Contributions

CL, TD, MG, and PB were involved in the conception and design of the study. CL, BS, MT, JW, AI, JG, and MG performed the data collection. CL, BS, MT, JW, and TD were involved in the data interpretation and analysis. CL, BS, PB, MG, and TD were involved in the writing and critical revision of the manuscript.

## Conflict of Interest Statement

The authors declare that the research was conducted in the absence of any commercial or financial relationships that could be construed as a potential conflict of interest.

## Footnotes

1 https://www.ncbi.nlm.nih.gov

2 https://www.ebi.ac.uk/Tools/msa/clustalo/

3 http://www.hiv.lanl.gov/content/sequence/ELIMDUPES/elimdupes.html
