# Supplementary material for: Geographical and Ethnic Differences Influence Culturable Commensal Yeast Diversity on Healthy Skin
Source: Front Microbiol. 2019 Aug 27;10:1891. doi: 10.3389/fmicb.2019.01891 (PMC6736582; doi:10.3389/fmicb.2019.01891)
Supplement: Supplementary file 1 [file Data_Sheet_1.docx]

Supplementary Material

Geographical and ethnic differences influence culturable commensal yeast diversity on healthy skin

Cheryl Leong^1^, Bettina Schmid^2^, Min Jet Toi^1^, Joyce Wang, Antony Sagayaraj Irudayaswamy^1^, Joleen Peh Zhen Goh^1^, Philipp P. Bosshard^2^, Martin Glatz^2^, Thomas L. Dawson, Jr^1,3*^

*^1^Skin Research Institute of Singapore, Agency for Science, Technology and Research (A*STAR), Singapore, Singapore****,*** *^2^Department of Dermatology, University Hospital Zurich, Zurich, Switzerland and Faculty of Medicine, University of Zurich, Zurich, Switzerland, ^3^Center for Cell Death, Injury & Regeneration, Departments of Drug Discovery & Biomedical Sciences and Biochemistry & Molecular Biology, Medical University of South Carolina, Charleston, SC*

*** Correspondence:**Thomas Dawson, Email: thomas_dawson@sris.a-star.edu.sg

**Supplementary Table 1.** LoD and LoQ of real-time qPCR primers

**Supplementary Figure 1.** Cohort size and composition

**Supplementary Figure 2.** Gender and race *Malassezia* species distribution in Singapore healthy skin isolates.

**Supplementary Figure 3.** Correlation plots representing the contribution of each factor to each respective dimension

**Supplementary Figure 4.** Species delineated factor maps for healthy skin isolates from Singapore and Zurich yeast species

**Supplementary Figure 5.** MCA plots and factor maps for comparison with other commensal yeasts

**Supplementary Figure 6**. ITS dendrograms for *M. sympodialis* (A) and *M. globosa* (B)

**Supplementary Table 1.** LoD and LoQ of real-time qPCR primers

| Species-specific primer | LoD | LoQ |
| --- | --- | --- |
| *M. restricta* | 4.01 | 9.68 |
| *M. globosa* | 2.77 | 6.63 |
| *M. sympodialis* | 4.10 | 16.93 |
| *M. slooffiae* | 23.44 | 29.51 |
| *M. furfur* | 1.56 | 5.20 |

**Supplementary Figure 1.**

**
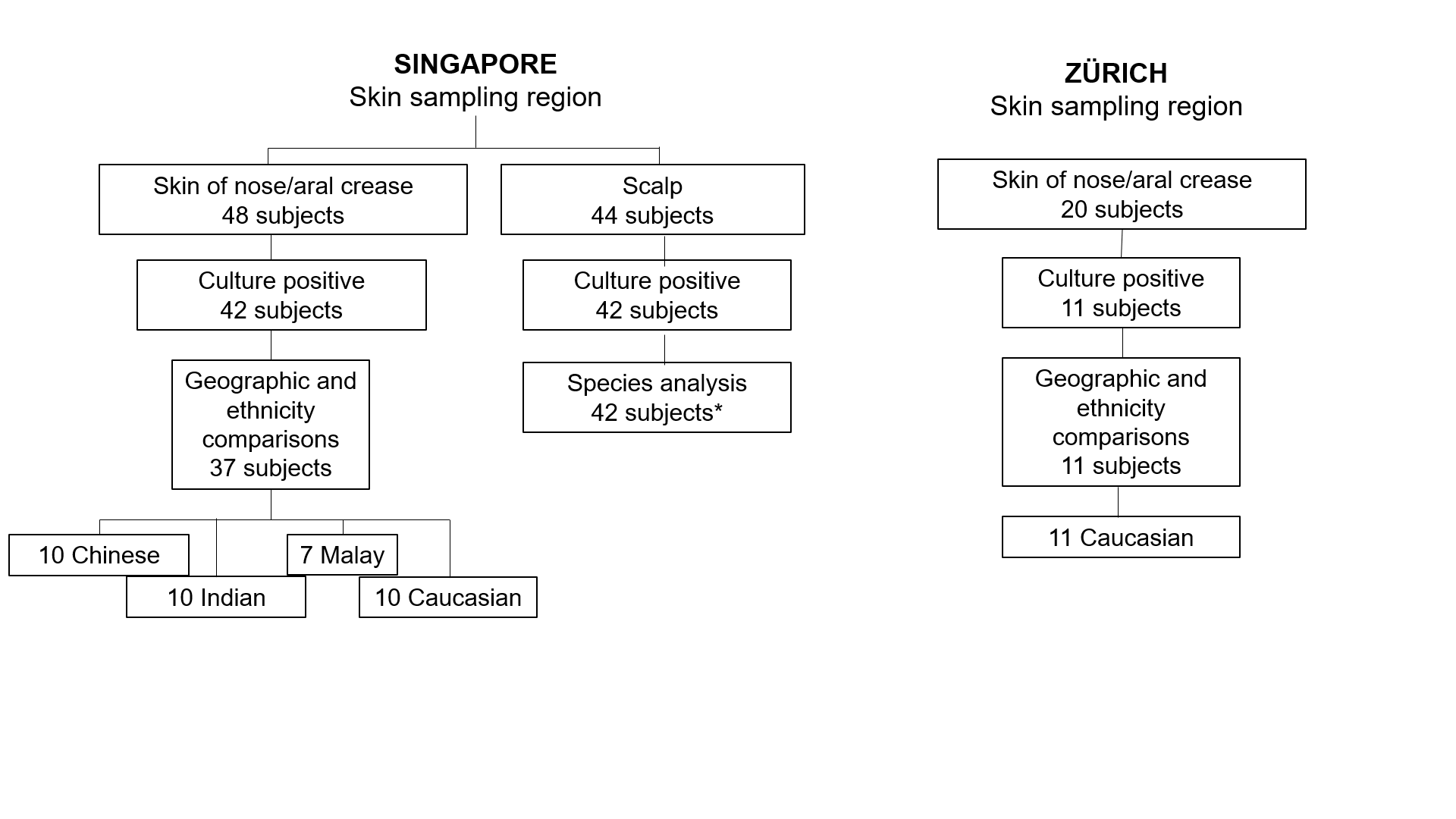
**

**44 subjects sampled on the scalp were not segregated by ethnic group

**Supplementary Figure 1.** Cohort characteristics for healthy subjects sampled from Singapore and Zürich.

**Supplementary Figure 2.**


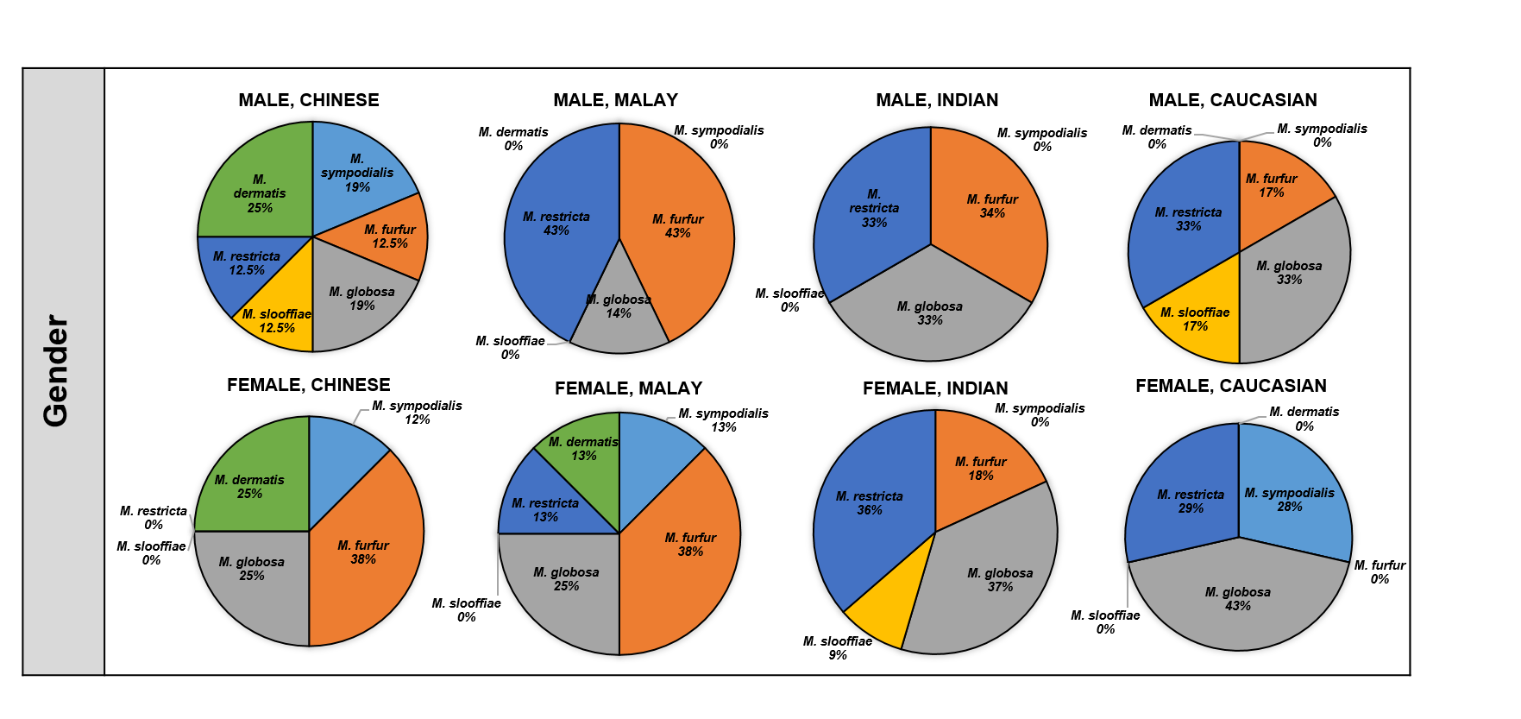


**Supplementary Figure 2.** Gender and race *Malassezia* species distribution in Singapore healthy skin isolates.

**Supplementary Figure 3.**

**Singapore only**

**Including other yeast species**

**Singapore only**

**Age comparison**

**Singapore vs Zurich**


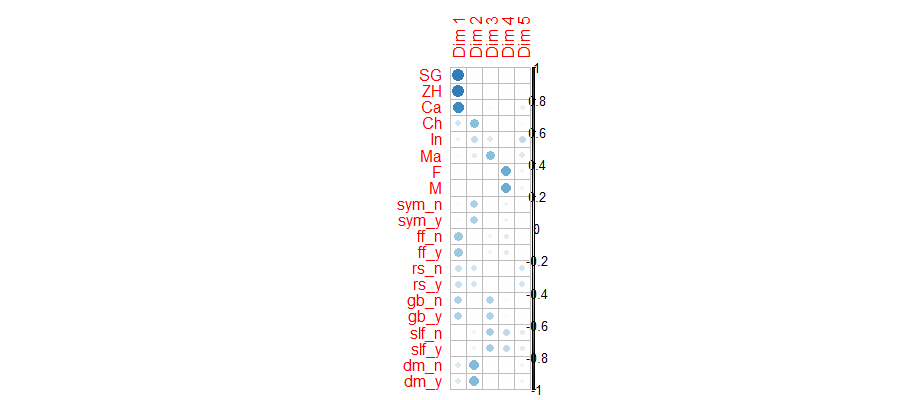

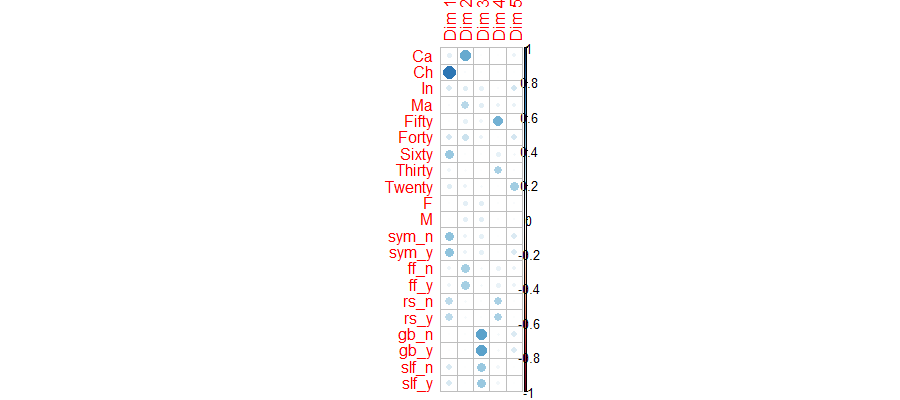

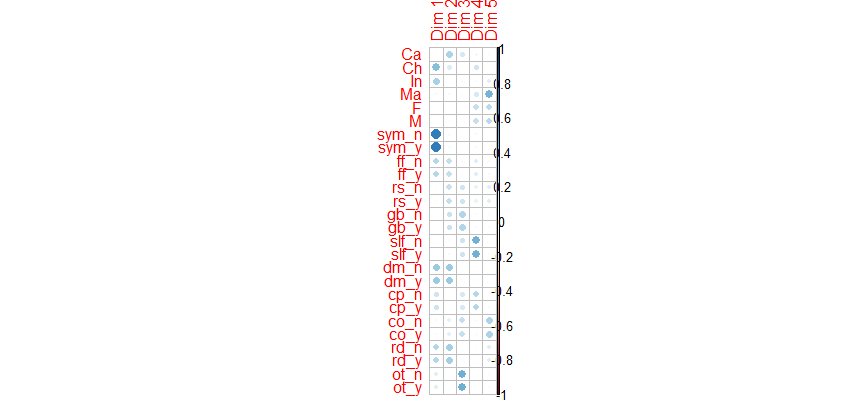


The contribution of each factor/variable category (in %) to each dimensions can be represented as above. Factors compared in different data set comparisons include gender (M-Male, F-Female), race (Ch-Chinese, Ma- Malay, Ind- Indian, Ca-Caucasian) and yeast species (CO-*Candida orthopsilosis*, CP-*Candida parapsilosis*, FF- *M. furfur*, GB- *M. globosa*, RD – *Rhodotorula*, SLF – *M. slooffiae*, SYM – *M. sympodialis*).

**Supplementary Figure 4.**


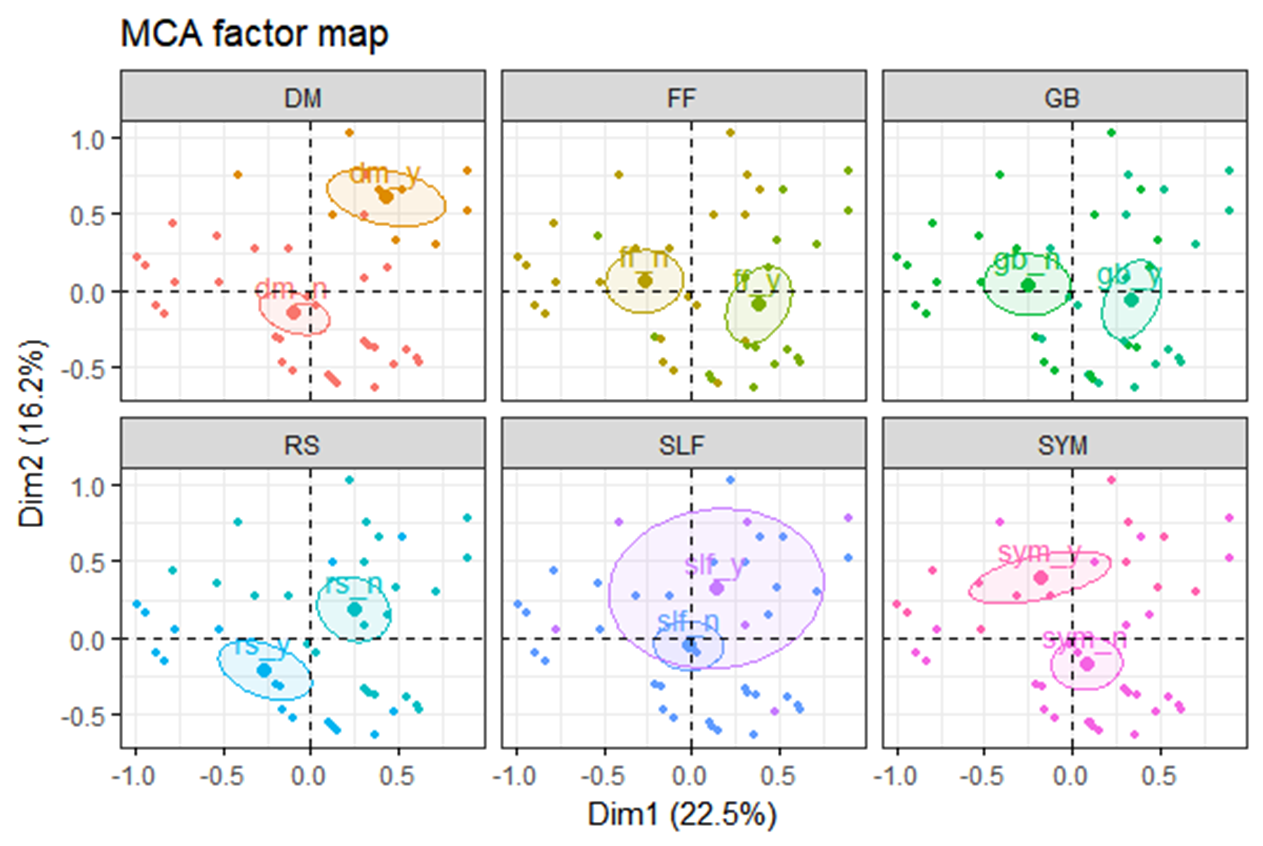


MCA plots and factor maps by *Malassezia* species (DM*- M. dermatis*, FF- *M. furfur*, GB- *M. globosa*, SLF – *M. slooffiae*, SYM – *M. sympodialis*) as defined by a 95% confidence ellipse) for healthy skin isolates from 37 individuals in Singapore.

**Supplementary Figure 5.**


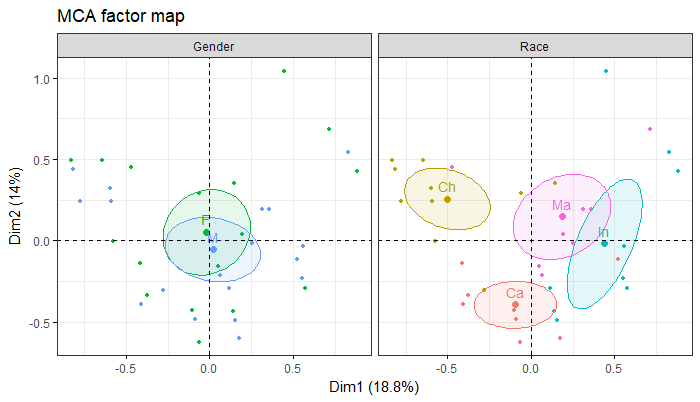


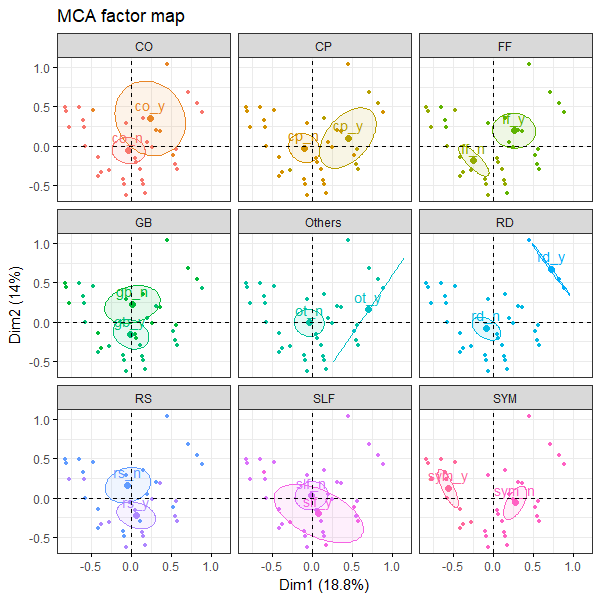


MCA plots and factor maps by gender (M-Male, F-Female), race (Ch-Chinese, Ma- Malay, Ind- Indian, Ca-Caucasian) and yeast species (CO-*Candida orthopsilosis*, CP-*Candida parapsilosis*, FF- *M. furfur*, GB- *M. globosa*, RD – *Rhodotorula*, SLF – *M. slooffiae*, SYM – *M. sympodialis*) as defined by a 95% confidence ellipse) for healthy skin isolates from 37 individuals in Singapore.

**Supplementary Figure 6.**

**A**


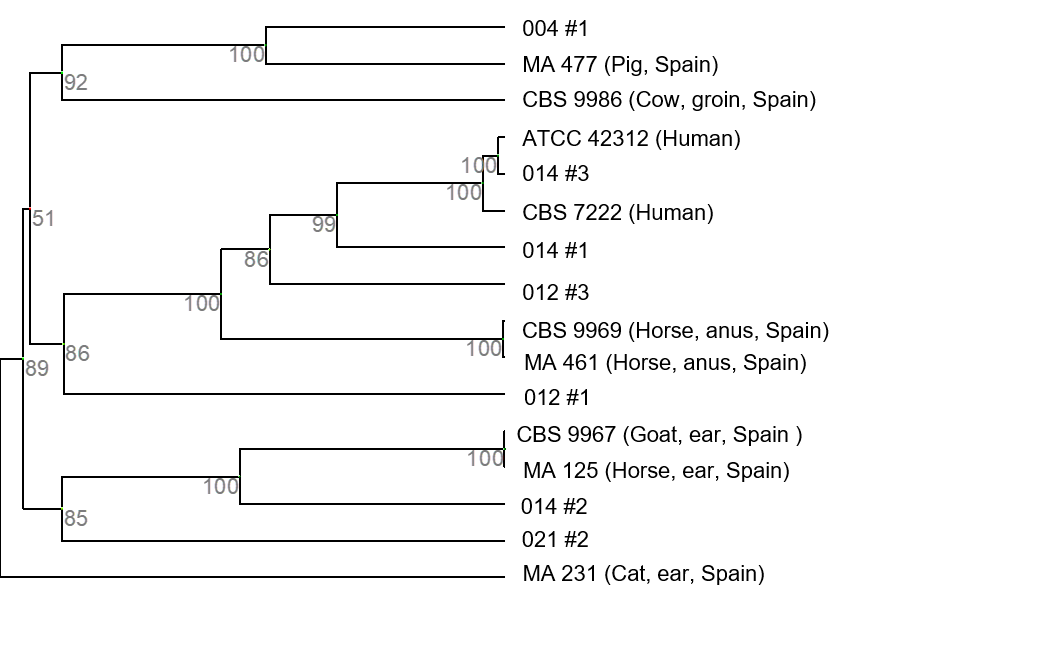


Unrooted dendrogram derived from 16 unique ITS sequences from *M. sympodialis*. All numbered isolates are derived from healthy human skin in Singapore unless indicated otherwise. Source information for strains from Spain were derived from F. J. Cabañes et al. 2005. Sequences not represented by any known reference database ID are indicated by their subject/strain codes.

**Supplementary Figure 6.**


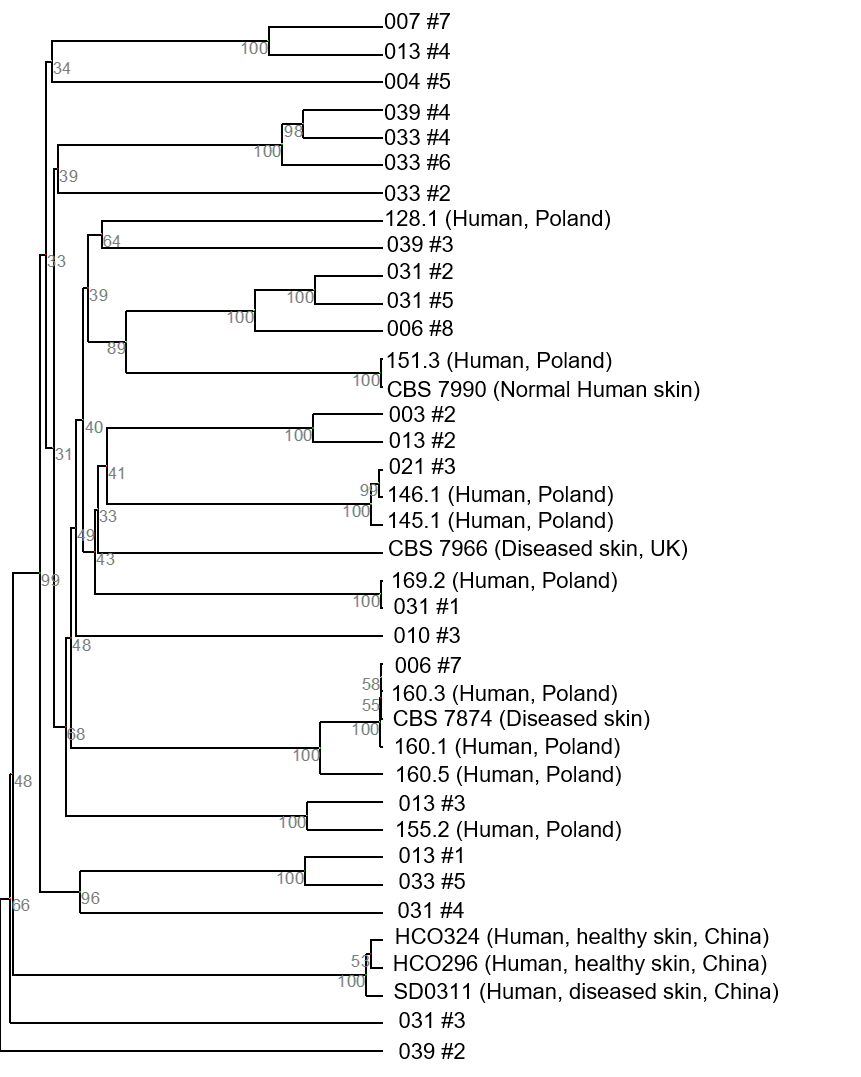


**B**

Unrooted dendrogram derived from 38 unique ITS sequences from *M. globosa*. All numbered isolates are derived from healthy human skin in Singapore unless indicated otherwise. Source information for strains from Poland and China were derived from Jagielski et al. 2014 and Lian et al. 2014 respectively. Sequences not represented by any known reference database ID are indicated by their subject/strain codes.
